# pH Gradient Liposomes Extract Protein Bound Amitriptyline in Peritoneal Dialysis—Exploratory Work

**DOI:** 10.3390/ijms231911577

**Published:** 2022-09-30

**Authors:** Grant Cave, Rachel Kee, Martyn Harvey, Zimei Wu

**Affiliations:** 1 Intensive Care Unit, Hawkes Bay District Health Board, Hastings 9014, New Zealand; 2 Waikato Hospital Emergency Department, Waikato District Health Board, Hamilton 3240, New Zealand; 3 Faculty of Medicine and Health Sciences, School of Pharmacy, University of Auckland, Auckland 1023, New Zealand

**Keywords:** liposomes, poisoning, peritoneal dialysis

## Abstract

Poisoning is a significant cause of injury-related death worldwide. Dialysis is usually ineffective in removing the toxin once it has been absorbed because of drug protein binding and high volumes of distribution. In this work, we explore whether the addition of liposomes to peritoneal dialysate could extract protein bound amitriptyline. Liposomes were prepared using the thin film hydration method. In the in vitro experiment, 3 mL of 20% albumin with a concentration of 6000 nmol/L amitriptyline in a proprietary dialysis cartridge was dialysed against 125 mL of phosphate-buffered saline with and without 80 mg 1,2-dioleoyl-sn-glycero-3-phosphoglycerol (DOPG) liposomes. In the in vivo arm, peritoneal dialysis was undertaken in 6 rats with pH gradient liposome augmented dialysate after intravenous amitriptyline injection. Peritoneal blood flow was estimated by CO_2_ extraction. Total amitriptyline extracted was compared to freely dissolved (non-protein bound) and total amitriptyline perfusing the membrane during the peritoneal dwell. Mean liposome size for DOPG and acidic centre pH gradient liposomes was 119 nm and 430 nm, respectively. In the in vitro experiment, more amitriptyline was extracted into the liposome containing dialysate than the control dialysate (40 +/− 2 nmol/L vs. 27 +/− 1 nmol/L). In the in vivo experiment, the total amitriptyline in dialysate was 5240 +/− 2750 nmol. Mean total free amitriptyline perfusing the peritoneal membrane was 93 +/− 46 nmol. Mean total blood amitriptyline perfusing the peritoneal membrane was 23,920 +/− 6920 nmol. Two of the six animals were excluded due to overestimation of peritoneal blood flow. This exploratory work suggests the addition of liposome nanoparticles to peritoneal dialysate extracted protein bound amitriptyline from blood.

## 1. Introduction

Poisoning is a significant cause of injury related death worldwide [1,2]. There are few specific antidotes, and treatment often consists of supportive care while toxins are cleared endogenously. Extracorporeal treatments in poisoning involve removing blood from the body, then applying a therapy to augment removal of the toxin from the externalised blood. While such therapies are theoretically attractive, it is not usually possible to meaningfully augment removal with extracorporeal therapy once a toxin has been absorbed. Currently, extracorporeal treatments are used in only 0.1–0.5% of exposures in the United States [3,4]. The most commonly used extracorporeal therapy is haemodialysis, where blood is dialysed in an extracorporeal circuit across a semipermeable membrane. Haemodialysis is recommended for only a few specific intoxications [5], and peritoneal dialysis is not recommended for any intoxications. Dialysis is explicitly proscribed in tricyclic antidepressant intoxication [6].

Two main factors limit the usefulness of dialysis in poisoning. Firstly, blood concentrations of toxins are often low relative to whole body toxin load. This means neither the extracorporeal dialysis circuits nor the peritoneal membrane cannot remove meaningful amounts of toxin at the blood flows presented to them. The volume of distribution of a drug or toxin is the apparent volume a given amount of a drug is dissolved in, given its blood concentration; the higher the volume of distribution, the less of that drug is in blood relative to the total body load. Dialysis is suggested to be ineffective for intoxication for toxins with volumes of distribution greater than 2 L per kilogram of body weight [7].

Secondly, free concentrations in blood are frequently low relative to total blood concentration due to blood protein binding. During dialysis only freely dissolved toxin can move across the dialysis membrane. Protein binding of toxin in blood reduces the freely dissolved concentration and decreases the usefulness of dialysis. Dialysis is suggested to be ineffective when >80% of the toxin in blood is bound to proteins [7].

Preclinical investigations have evaluated three strategies to mitigate the effect of protein binding in poisoning:

(a) Introducing a nanoparticle toxin scavenger into dialysate. This reduces dialysate toxin concentrations, thus maintaining the gradient across the membrane, driving toxin removal [8].
(b) Introducing a competitor for blood protein binding sites, often at the point of blood flowing into the dialysis circuit. This increases free toxin concentrations on the blood side of the dialysis membrane, increasing the gradient driving removal [9].
(c) Increasing the dialysate flow rate relative to blood flow rate haemodialysis circuits. This reduces dialysate toxin concentrations thus maintaining the gradient across the membrane, driving toxin removal.

This paper presents exploratory work to investigate whether the first of these strategies could mitigate the effect of protein binding for a highly bound toxin using amitriptyline as a model drug. Amitriptyline was chosen as it has a high volume of distribution, is highly bound to protein in plasma [10], and as a lipophilic weak base is sequestered effectively both by acidic centre pH gradient liposomes and liposomes which bind by electrostatic interaction in the membrane.

### 1.1. Liposomes

**Figure 1:**
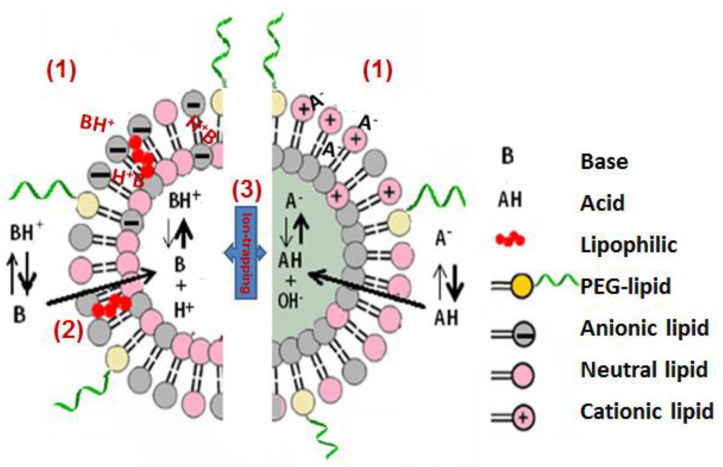
Mechanisms by which liposomes interact with drugs of varied physicochemical properties: (**1**) electrostatic interactions, (**2**) lipid-lipid interaction, and (**3**) ion-trapping of lipophilic weak bases or acids driven by transmembrane pH-gradients. Reproduced with permission from Ref. [11]. Copyright 2018 Elsevier Inc. Cambridge, MA, USA.

Liposomes are nano-sized spherical vesicles of phospholipid bilayer with aqueous cores. Liposomes have been widely used in the pharmaceutical industry for drug delivery. The use of liposomes as agents of detoxification is a modification of this traditional use. Rather than being loaded with drug in vitro for drug delivery, administration of in vitro “empty” liposomes aims to create an avid toxin binding sump. Liposomes can to bind the toxin through lipophilic or electrostatic interactions in the membrane, or in a pH-controlled liposome core via the ion trapping phenomenon whereby the extra-liposomal unionised drug molecules can enter the liposomes and become entrapped in the aqueous centre. For a weakly basic drug, once in the liposome core with a low pH the drug will become ionised and thus cannot diffuse out from the lipid bilayers. Polyethylene glycol is often attached to a small proportion of phospholipids to decrease protein binding in vivo. These mechanisms are demonstrated in Figure 1 [11].

### 1.2. Liposomes in Dialysis

Protein bound uraemic toxins (PBUTs) are gastrointestinal bacterial metabolites of tyrosine and tryptophan that accumulate in patients treated with long term dialysis for renal failure. Dialysates containing liposomes have been demonstrated to extract PBUTs from binding proteins in blood and modelled blood compartments [8].

Liposome supported peritoneal dialysis (LSPD), where peritoneal dialysate is enriched with acidic centre pH gradient liposomes, has ameliorated poisoning in several animal models [12,13]. Markedly increased dialysate drug concentrations for LSPD compared with non-augmented peritoneal dialysate in rat models have been demonstrated for haloperidol, verapamil and amitriptyline [12,13]. Previously estimated median extraction rate for amitriptyline from blood into LSPD dialysate is 33% in a rat model [13], significantly more than the reported single figure free fraction of amitriptyline in blood. This work generated the hypothesis explored in this experiment.

### 1.3. Hypotheses Explored in This Work

A peritoneal dialysis model was used to further this hypothesis both as it was the model used in our initial work and the proximity of liposome augmented peritoneal dialysis to clinical use [14]. First, an in vitro experiment with liposome enriched dialysate measuring total and free drug concentrations was used to evaluate feasibility, prior to an animal experiment. Next, a rat peritoneal dialysis experiment was adopted, where CO_2_ clearance into dialysate was used to estimate peritoneal blood flow. If the product of free drug concentration and peritoneal blood flow is less than the amount extracted into peritoneal dialysate, then protein bound drug must have been extracted. A calculation of the percentage of the total drug delivered to the peritoneal membrane extracted into dialysate can also be made.

## 2. Results

### 2.1. Liposome Characteristics

After manufacture, the mean acidic centre pH gradient liposome size was 430 nm with a polydispersity index (PDI) of 0.48 and a mean zeta potential of −14.5 millivolts (mV). The mean DOPG liposome size was 119 nm, with a PDI of 0.1 and a mean zeta potential of −33.0 mV.

### 2.2. In Vitro Experiment

Concentrations were measured 3 times for each sample. The initial concentration of amitriptyline in 3 mL of 20% albumin was 6007 +/− 551 nmol/L. Free concentration in 3 mL 20% albumin was 160 +/− 10 nmol/L.

More amitriptyline was extracted into the liposome containing dialysate than the control dialysate. The free concentration of amitriptyline in the liposome containing dialysate was approximately half that of control. Concentration at the end of the dwell in control dialysate was 27 +/− 1 nmol/L. Concentration at the end of the dwell in liposome augmented dialysate was 40 +/− 2 nmol/L. A further 10% of the 18 nmol of amitriptyline placed in the dialysis cartridge was extracted into liposome enriched dialysate, relative to control. Free concentration in liposome augmented dialysate was 14 +/− 1 nmol/L.

**Figure 2:**
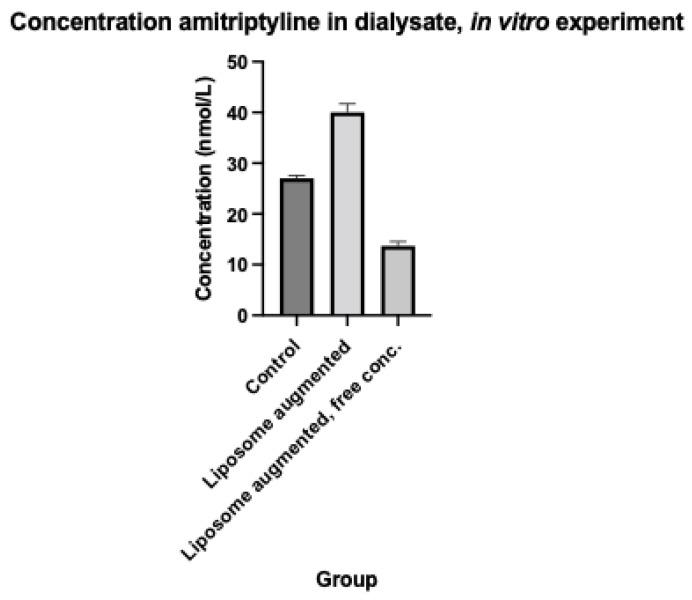
Amitriptyline concentrations by group, in vitro experiment.

These results are demonstrated graphically in Figure 2.

### 2.3. In Vivo Experiment

**Figure 3:**
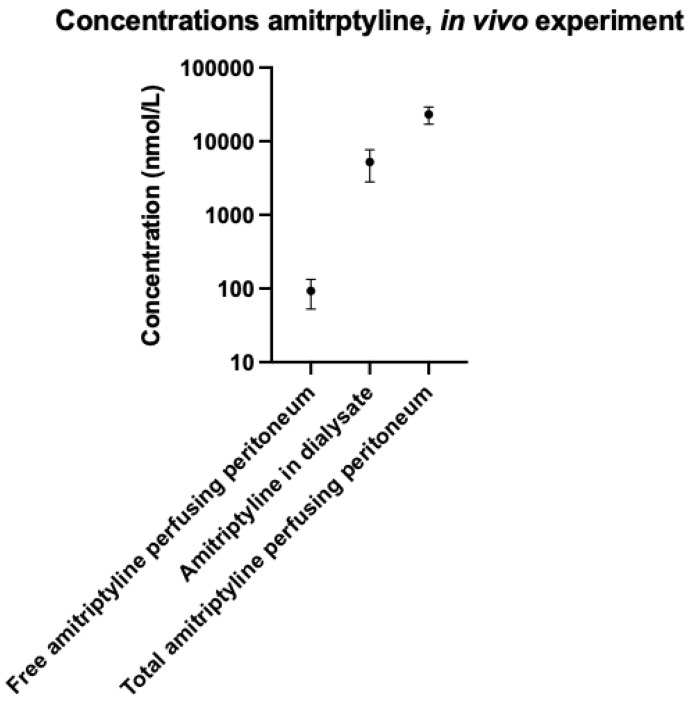
Concentrations of amitriptyline in dialysate against free and total amitriptyline perfusing the peritoneal membrane.

2 animals were excluded based on estimated PBF > 5.4 mL/min. For the remaining 4 animals, mean PBF was measured as 3.6 +/− 1.2 mL/min. After five minutes, a greater amount of amitriptyline was in dialysate, compared to freely dissolved amitriptyline perfusing the peritoneal membrane. The total amitriptyline in dialysate was 5240 +/− 2750 nmol. Mean total free amitriptyline perfusing the peritoneal membrane was 93 +/− 46 nmol. Mean total blood amitriptyline perfusing the peritoneal membrane was 23,920 +/− 6920 nmol. These concentrations are shown graphically in Figure 3. The mean percentage of total amitriptyline perfusion to the peritoneal membrane that was extracted into dialysate was 23.3 +/− 13.1%.

## 3. Discussion

Our results represent promising exploratory work suggesting protein bound toxins may be made more dialysable by introducing an appropriate nanoparticle binder in dialysate. To move a bound toxin from the blood compartment into a binding nanoparticle in dialysate, it must first dissociate from blood protein. Then, in the freely dissolved phase in blood, the toxin must move down a concentration gradient across the dialysis membrane into the freely dissolved phase in dialysate. Throughout this process, the dialysate free toxin concentration is maintained at a low level as it binds to nanoparticles in dialysate [15]. Our results support this sequence on two points. In the in vivo experiment, a much greater amount of amitriptyline was extracted into dialysate than simply perfused the dialysis membrane as freely dissolved amitriptyline. This is consistent with protein bound amitriptyline (the great majority of amitriptyline perfusing the membrane) in blood dissociating from proteins, then moving across the peritoneal membrane into dialysate. In the in vitro experiment, more amitriptyline was extracted into liposome enriched dialysate, while the freely dissolved amitriptyline concentration in dialysate was lower relative to control, consistent with liposome enrichment decreasing free dialysate concentrations. 

Liposome enrichment of dialysate is a growing field. In poisoning, previous work has demonstrated improved blood pressure and verapamil extraction in verapamil toxic rats managed with liposome enriched peritoneal dialysate [12]. No estimation of whether verapamil extraction exceeded free toxin delivery to the peritoneal membrane was made in this study. Liposome enrichment has increased dialytic extraction of the endogenous protein bound uraemic toxins which accumulate in renal failure in both in vitro and in vivo models [15,16,17,18]. Clinical study is most advanced in the management of hyperammonaemia secondary to liver failure. The use of acidic centre pH gradient liposomes to extract ammonia into peritoneal dialysate in this setting is currently in phase 2 studies [14]. Developments in the use of liposomes for this nascent indication may precipitate further work into potential use in poisoning. 

While these results are positive with for the limitation of protein binding on dialysis, there is no mitigation on the limitation of high volume of distribution. Attempts by our group to mitigate this limitation by introducing toxin binders into the vascular space have thus far been unsuccessful [19]. Further work in this regard is planned.

Our work is preliminary and holds many limitations. Small numbers in the in vivo arm and a small number of observations in the in vitro experiment make these findings exploratory rather than hypothesis confirming. Numbers were small in the in vitro experiment as we wished only to confirm presence of a signal before progressing to in vivo work. We initially planned to show a large effect and report a *p* value with the in vivo work. Excluding subjects based on overestimates of peritoneal blood flow led to a reduction in numbers and confidence interval reporting being more appropriate.

Our method to calculate peritoneal blood flow has a systematic tendency to overestimate, given the potential for anaerobic CO_2_ production from bicarbonate in dialysate. We excluded values for PBF > 100% over those previously described and accepted other values on the basis that an overestimate of PBF would bias against the effect of interest. Different liposomes were used in the in vitro and in vivo experiments. While acidic centre pH gradient liposomes have been demonstrated to have a higher binding capacity for amitriptyline [12], the long dwell time in the in vitro experiment was thought to potentially degrade this binding capacity. DOPG liposomes which bind amitriptyline via electrostatic attraction in the membrane [20] were thus used. While there was no control arm to liposome enriched dialysate in the in vivo experiment, liposome augmented dialysates have previously been demonstrated increase amitriptyline extraction [12]. Much longer dwell times are used clinically for peritoneal dialysis than in the present experiment. Any future work may use longer dwell times, particularly given the potential for saturation of liposome toxin binding.

## 4. Materials and Methods

### 4.1. Liposome Preparation

1,2-dioleoyl-sn-glycero-3-phosphoglycerol (DOPG) liposomes and acidic centre pH gradient liposomes were prepared using the thin film method as described previously [13]. In brief, phospholipids and cholesterol were dissolved in an organic solvent, then this solvent was evaporated off under hypoxic conditions. The resultant thin film was then hydrated in an aqueous medium. The tendency of the system toward lowest Gibbs free energy results in the formation of spherical bilayers of phospholipid in suspension. The suspension was then ultrasounded and filtered to yield liposomes of an appropriate size. Liposome size and charge were measured using a Malvern Nano ZS Zetasizer, (Malvern Instruments, Malvern, UK).

### 4.2. In Vitro Experiment

3 mL of 20% albumin with an estimated concentration of 6000 nmol/L amitriptyline was placed in a proprietary dialysis cartridge (Slide-A-Lyzer, Thermo Fisher Scientific, Waltham, MA, USA) which was then placed in a 125 mL of phosphate-buffered saline (control) or 125 mL phosphate-buffered saline with 80 mg DOPG liposomes. Dwell time was 12 h. DOPG liposomes were chosen as the electrostatic mechanism via which these liposomes bind amitriptyline, as it would not be expected to degrade over the dwell time. At the end of the dwell, total and unbound concentrations were measured in both the cartridge and liposome augmented dialysate. Total concentration only was measured in control dialysate.

### 4.3. In Vivo Experiment

#### 4.3.1. Animals

6 Female Sprague Dawley rats were studied with an age range of 119–222 days and weight range 310–435 g. Animals were kept in single gender enclosures with no chance of pregnancy. Twelve-hour light-dark cycles (lights on/off at 07:00/19:00 h) and climate control were maintained. Access to feed and water was allowed ad libitum until the day of animal utilization.

#### 4.3.2. Animal Manipulations

Ethical approval for all animal manipulations was given by the Ruakuara Animal Ethics Committee. On the day of study animals were sedated with ketamine at 50 mg/kg (Mayne Pharma Ltd., Auckland, New Zealand), and xylazine at 4 mg/kg (Bayer HealthCare, Leverkusen, Germany) via intraperitoneal injection. Animals were then placed on a warming board at 38.5 °C. An intravenous cannula was placed in the tail vein using a needle over catheter technique (24G × 0.75 in Insyte, BD, Switzerland). Following dissection of the anterior neck, a tracheostomy tube (14G × 1.77 inch Insyte, BD, Switzerland) was placed under direct vision. Mechanical ventilation using a small animal ventilator (Inspira ASV, Harvard Apparatus, Holliston, MA, USA) was then undertaken with oxygen as the inspired gas admixed with 2% isoflurane (Merial, Auckland, New Zealand) via a vaporizer (Somnosuite Small Animal Anesthesia System, Kent Scientific Corporation, Torrington, CT, USA) to maintain anaesthesia. ECG electrodes (MLA1213 Needle Electrodes, AD Instruments, Bella Vista, Australia) were placed in 3 limbs of the rat for continuous ECG monitoring (Animal Bio Amp, AD Instruments, Bella Vista, Australia). 

A carotid arterial line was placed under direct vision using a catheter over needle technique and tied off proximally and distally (24G × 0.75 inch Insyte, BD, Switzerland) allowing for arterial blood sampling and monitoring of arterial pressure (BP Amp, AD Instruments, Bella Vista, Australia). Anaesthesia, mechanical ventilation, and BP and ECG monitoring were continued for the duration of the experiment. 

A 1 cm incision was made in the ventral midline 2 cm below the xiphisternum for placement of a peritoneal dialysis catheter. The abdominal muscles were grasped and elevated with subsequent dissection into the peritoneal space. A 10 cm fenestrated catheter was directed into the right lower quadrant and the incision in the abdominal wall, then the peritoneal space was sealed via a clamp over the incision.

### 4.4. Experimental Protocol

Following completion of animal manipulations and collection of baseline metrics amitriptyline (Sigma Aldrich, Sydney, Australia) dissolved in 0.9% NaCl (2.5 mg/mL) was injected into the tail vein over 2.5 min at a dose of 5 mg/kg. The end of amitriptyline injection was nominated time 0 (T0). 

At 10 min following drug administration (T10) blood was drawn for arterial blood gas evaluation, and blood amitriptyline concentration. Simultaneously 20 mL of dialysate/liposome suspension was injected into the peritoneal cavity over 30 s and manually agitated.

At T 15 min (T 15) a further blood sample was drawn for arterial blood gas analysis, and blood amitriptyline concentration measurement. Peritoneal dialysate was likewise drawn for measurement of PCO_2_ and dialysate amitriptyline concentration.

After the manipulations undertaken at T15, mechanical ventilation was ceased and a terminal bleed was taken from the carotid arterial line. Death was confirmed by absent respiratory efforts and examination of the ECG trace. 

### 4.5. Amitriptyline Assay/CO_2_ Measurements

Amitriptyline concentrations were measured using an LCMS method (Sciex 3200 MSD Aglient 1200 series LS, Sciex, (Redwood City, CA, USA)) with isotope matched internal standards. The whole blood sample was prepared by a protein crash with acetonitrile, followed by centrifugation after which an aliquot was presented to the instrument for analysis. A four-point matrix-matched standard curve was used to calibrate the analyser for each run with two levels of quality control. The coefficient of variation on this measurement was 6.6% and the assay had linear correlation with amitriptyline concentration up to 40,000 nmol/L. Free fraction was measured as that in solution after centrifugal ultrafiltration of a pre-acetonitrile sample for 30 min at 37 degrees Celsius at 2000× *g*. The exclusion membrane was rated to exclude molecules >30,000× *g*/mole. 

Blood and peritoneal partial pressure of CO_2_ was measured using the iSTAT Alinity device and CG4+ cartridges (Abbott Point of Care, Princeton, NJ, USA).

### 4.6. Measurement of Peritoneal Blood Flow

Carbon dioxide (CO_2_) clearance into peritoneal dialysate over the initial 5 min of dialysis has been demonstrated to have a correlation with other measures of peritoneal blood flow (PBF) in the rat model [21]. It was assumed there was no change in dialysate volume (20 mL) over the 5 min dwell period, and that all CO_2_ present in dialysate was the result of transfer of dissolved CO_2_ across the peritoneal membrane. Equation (1) is taken from Grzegorzewska et al. and was used to calculate peritoneal blood flow.

PBF = (volume in dialysate/time) × [ln (mean blood pCO_2_) − ln (mean blood pCO_2_ − dialysate pCO_2_ at 5 min)] (1)

A limitation of using pCO_2_ to calculate PBF is the potential for overestimation when bicarbonate diffusing into dialysate is metabolised to CO_2_. While reported not to occur if dialysate pH is >7, Grzegorzewska noted evidence of anaerobic CO_2_ production over longer dwells, with dialysate pCO_2_ exceeding that of blood in some subjects. Given the short dwell time we chose to deal with this by excluding subjects where the estimation of PBF was >5.4 mL/min, greater than twice that measured in previous work. 

Total and free amitriptyline delivery were calculated as the mean total and free blood concentrations over the time of the peritoneal dwell multiplied by PBF. Total amitriptyline in dialysate was calculated as the end concentration in dialysate times dialysate volume. 

### 4.7. Statistics

This is exploratory work on small numbers and as such no *p* values are reported. Values are reported as means +/−95% confidence intervals.

## 5. Conclusions

Exploratory work suggests addition of liposome nanoparticles to dialysate extracted protein bound amitriptyline from blood into peritoneal dialysate.

## Data Availability

Data are available from the corresponding author on reasonable request.
